# *Culex territans* mosquitoes as a vector of Giant Anuran Trypanosomes

**DOI:** 10.1186/s13071-026-07399-w

**Published:** 2026-04-21

**Authors:** Joanna Reinhold, Isabella Roeske, Iris E. Schmeder, Ella Halbert, David S. Mcleod, Chloé Lahondère

**Affiliations:** 1 Department of Biochemistry, Virginia Polytechnic Institute and State University, Blacksburg, VA 24061 USA; 2 The Fralin Life Science Institute, Virginia Polytechnic Institute and State University, Blacksburg, VA 24061 USA; 3 Center of Emerging, Zoonotic and Arthropod-borne Pathogens, Virginia Polytechnic Institute and State University, Blacksburg, VA 24061 USA; 4 Department of Environmental Sciences, Emory University, Atlanta, GA 30322 USA; 5 Department of Integrative Biology, University of Texas, Austin, TX 78712 USA; 6 Oberlin College, Oberlin, OH 44074 USA; 7 Department of Natural Sciences, Mary Baldwin University, Staunton, VA 24401 USA; 8 The Global Change Center, Virginia Polytechnic Institute and State University, Blacksburg, VA 24061 USA; 9 Department of Entomology, Virginia Polytechnic Institute and State University, Blacksburg, VA 24061 USA; 10 Present Address: Department of Biological Sciences, Longwood University, Farmville, VA 23901 USA

**Keywords:** Northern frog-biting mosquito, Anuran parasites, Green frogs, Bullfrogs, Disease ecology, Transmission

## Abstract

**Background:**

Amphibian populations are declining worldwide, in part due to diseases caused by viruses, fungi, andparasites. Giant Anuran Trypanosomes (GATs) are parasites that affect frogs worldwide and require a vector to betransmitted. *Culex territans* is an amphibian-feeding mosquito suspected to be a vector of trypanosomes, but this hasnot previously been confirmed.

**Methods:**

In this study, we tested blood-fed Cx. territans and blood from their primary anuran hosts, *Rana clamitans* and *R*. *catesbeiana*, in southwest Virginia. Additionally, we tested potential routes of transmission from the mosquito tothe frog.

**Results:**

We found trypanosomes present in both mosquitoes and anurans and found trypanosomes present in thefeces 2 days after being blood fed on infected frogs, as well as in the body and saliva 14 days post-feeding.

**Conclusions:**

Overall, this study contributes to our knowledge of the GAT epidemiology and the role Cx. territans mightplay in their transmission.

**Graphical Abstract:**

**Figure:**
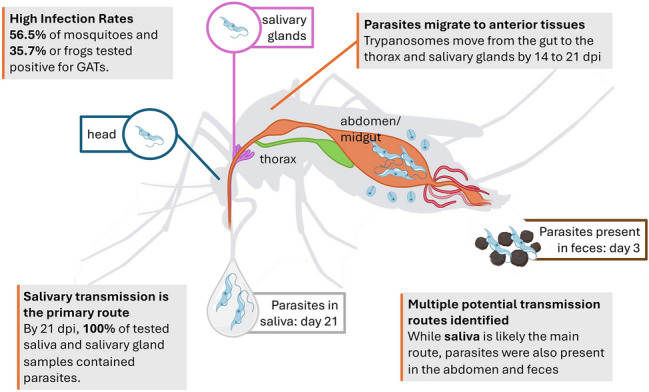

**Supplementary Information:**

The online version contains supplementary material available at 10.1186/s13071-026-07399-w.

## Background

Among vertebrates, amphibians are most at risk of population declines and extinctions due to many factors, including climate change, habitat destruction, and diseases [1–4]. Certain pathogens, such as Ranaviruses, chytrid fungi, and parasites, have been implicated in population declines, mass die-offs, and total extinction events [2, 4–7]. Trypanosomes are protozoan blood parasites with a worldwide distribution that require an invertebrate vector to be transmitted to new vertebrate hosts [8]. In humans, trypanosomes can cause fatal diseases, such as Human African Trypanosomiasis (viz., “sleeping sickness”; *Trypanosoma brucei gambiense* and *T. brucei rhodesiense*) and Chagas disease (*T. cruzi*) [9]. Giant Anuran Trypanosomes (GATs), including *T. rotatorium, T. ranarum, T. fallisi*, and *T. mega*, are known to hemoparasitize frogs and toads and are found in North America and other continents [8, 10, 11]. The diversity of these parasites is poorly understood, and much work is still needed to evaluate infection rates and pathogenicity [10–13]. Studies on the pathogenicity of GATs are limited, but there is some evidence of lethal infections in frogs and tadpoles, particularly under conditions of high parasite load [14, 15]. The primary mode of transmission for these parasites is through invertebrate vectors, but the full range of potential vectors has not been determined. Leeches are known to transmit trypanosomes, including GATs, to fish and amphibians [16, 17], and a biting midge (the stone fly, *Corethrella wirthi)* is known to transmit trypanosomes to frogs [18]. It has been proposed that the northern frog-biting mosquito (*Culex territans*)*,* might also be involved in the trypanosome transmission cycle [8, 19]*.*

Mosquitoes in the *Culex* genus are competent vectors of several pathogens, including West Nile virus (WNV), Eastern equine encephalitis (EEE), Western equine encephalitis (WEE), and *Wucheraria bancrofti*, which they transmit through their saliva while biting/feeding [20–23]. In amphibians and reptiles, *Cx. territans* is a known vector of the parasitic roundworm *Foleyellides flexicauda* [24] and *Hepatozoon* parasites [25]. Desser et al. [19] showed that *T. rotatorium* can develop in *Cx. territans*, but its vectorial capabilities for trypanosomes remain unknown.

Parasites are released from their vectors via several mechanisms. *Hepatozoon* parasites are transmitted by *Cx. territans* to their anuran hosts via ingestion [25, 26]. By contrast, *T. cruzi* is transmitted from kissing bugs through contact with infected feces [27], whereas other trypanosomes (e.g., *T. b. rhodesiense, T. b. gambiense,* and *T. b. brucei*) are spread through biting [27]. The avian trypanosome, *T. culivavium*, adheres to the alimentary canal of *Cx. quinquefasciatus* and is likely transmitted through regurgitation [28, 29]. Many other mosquito species transmit pathogens that have migrated to the salivary glands and are injected in the host via their saliva while feeding [30].

In this study, we screened for GATs in *Cx. territans* mosquitoes as well as in their primary hosts, green frogs (*Rana clamitans*) and American bullfrogs (*Rana catesbeiana*), at a previously unsurveyed site in Virginia, and tested the vector competence of these mosquitoes for GATs by examining the saliva, feces, and body segments of blood-fed mosquitoes [31]. We hypothesized that *Cx. territans* mosquitoes can transmit these parasites to their hosts through biting. Understanding the prevalence and transmission mode of these anuran parasites provides a better insight into anuran disease ecology as well as the evolution of trypanosome transmission.

## Methods

### Mosquito collection

**Figure 1:**
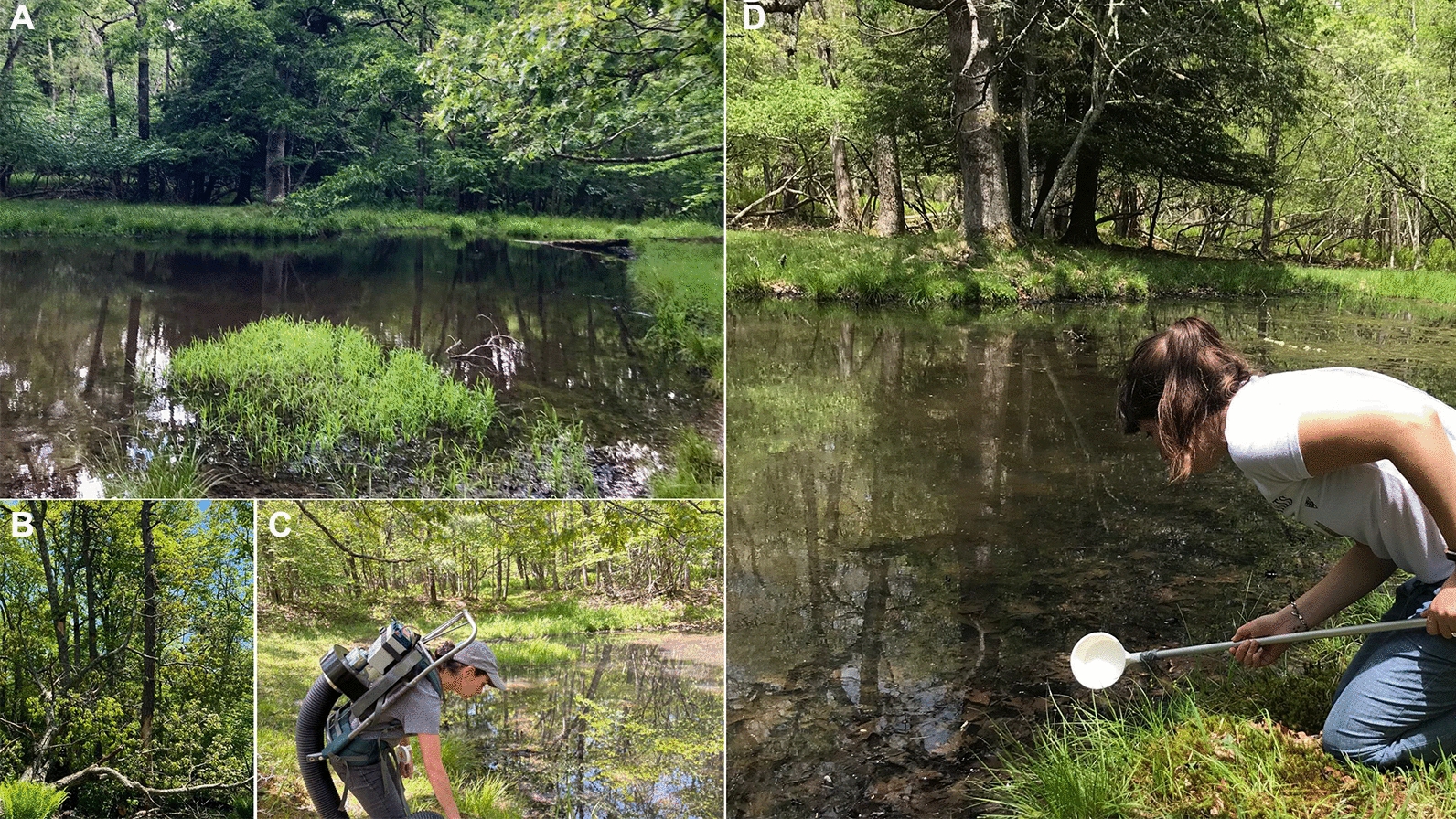
**A** Sylvatica Pond. **B** Horton Pond. **C** Adult mosquito collection using a CDC backpack aspirator. **D** Mosquito larvae and pupae collection via water scooping (Picture credit: A, B and D by Chloé Lahondère. C by Iris Schmeder)

Adult *Cx. territans* were collected from Sylvatica Pond (37.377079, −80.522245) at Mountain Lake Biological Station (MLBS), Giles County, Virginia, USA, (37.3746, −80.5214; 1160 m ASL) using a Centers for Disease Control and Prevention (CDC) backpack aspirator (John Hock Company, Gainesville, FL, USA) during the summer months (May–August) of 2021–2023 and 2025 (Fig. 1). Mosquitoes were not collected for this study in 2024 due to an interruption in study operations. Blood-fed and gravid females were identified on the basis of external morphology [32] and stored at −80 °C until testing. In 2021, blood-fed mosquitoes were dissected to separate the abdomen (containing the blood meal) from the head and thorax (anterior tissues). This facilitated distinction between ingested trypanosomes present only in the blood meal and those trypanosomes disseminated from a prior blood meal into the anterior tissues of the mosquito. Gravid females from 2021 were processed to test for dissemination to determine whether trypanosomes would be present following digestion. Findings from 2021 informed our trypanosome surveillance strategy, in which only the blood meals from field-caught mosquitoes were tested in subsequent years.

Larval and pupal *Cx. territans* collected from Sylvatica Pond were used for transmission assays (Fig. 1). All mosquitoes were maintained in a climatic chamber at 26 ± 1 °C, 70% humidity, with a 14:10 light cycle. Larvae were released into a larval tray (BioQuip Industries, Rancho Dominguez, CA) with deionized water and fish food (Hikari First Bites powdered fish food, Kyori Food Industries, Japan). Pupae were separated into a funnel container (Small Berlese Funnel Trap, BioQuip), and 6 days after emergence, species identification was verified, and all unfed female *Cx. territans* were placed in separate containers for use in transmission assays.

### Frog collection

The taxonomy of true frogs (Genus *Rana*) has had a tumultuous recent history [33]. We take a conservative approach following the taxonomy of AmphibiaWeb for simplicity and coherence [34]. Adult *Rana clamitans* and *R. catesbeiana* were collected by hand and nets from Riopel (37.374552, −80.552109), Sylvatica, and Horton (37.378483, −80.522078) ponds at MLBS during the summers of 2022, 2023, and 2025. Permits were provided by Virginia Tech IACUC (#19-003, #22-060, and #25-076), the United States Department of Agriculture (USDA), and the Virginia Department of Wildlife Resources. Frogs were captured from 20:00 to 23:00 and kept in polyethylene bags (10 × 16 in 4 Mil Industrial Poly Bags, ULINE, Pleasant Prairie, WI) with spring water. Larger frogs were kept in terrariums (23 cm × 15 cm × 18 cm Kritter Keeper, Lee’s Aquarium and Pet Products, San Marcos, CA, USA), which were bleached between uses to prevent cross-contamination. All frogs were held for less than 24 h and subsequently released at the point of capture. Recapture within 3 days was avoided by marking and identifying individuals via toe clipping [35]. Frog blood was sampled from the musculocutaneous vein using methods described in Forzan et al. [36]. Samples were stored at −80 °C until testing.

### Trypanosome transmission

After finding evidence of dissemination in field-caught mosquitoes, we determined transmission capacity using 20 female *Cx. territans* mosquitoes that were blood fed at one time on a single male *R. clamitans*, and polymerase chain reaction (PCR)-confirmed as naturally positive for trypanosomes (likely *T. ranarum*), as described below. Mosquitoes were housed in fly tubes (*Drosophila* vials, Stellar Scientific, Baltimore, MD 21209) with wetted filter paper at the base for humidity and mesh at the top, providing ad libitum access to a cotton ball soaked in 10% sucrose until they defecated. Feces were collected from the tube after 3 days and extracted from the filter paper by rinsing it with PBS. The samples were then tested for the presence of trypanosomes using PCR. After 14–21 days post-infection (dpi), mosquitoes were cold-anesthetized and tethered to a tungsten rod using ultraviolet (UV)-cured glue (Bondic, Niagara Falls, NY, USA) and mounted so that they were held in one position for forced salivation. The proboscis of each mosquito was inserted into a 1 mm diameter capillary tube (QF100-70-10, Sutter Instruments, Novato, CA, USA) filled with a 1:1 solution of fetal bovine serum (FBS, Thermofisher, Waltham, MA, USA) and 10% sucrose. Each mosquito was given 30 min to feed/salivate. The solution containing the saliva was then screened for trypanosomes using DNA extraction and PCR (as described below). Following feeding, the salivary glands of each tethered mosquito were dissected to screen for the presence of trypanosomes. During dissection, the proboscis and salivary glands were removed using forceps, the head was removed from the body using a disposable blade, and the thorax and body were separated. Each body part was screened separately for the presence of trypanosomes using DNA extraction and PCR.

### DNA extraction, PCR, and Sanger sequencing

DNA from both frog blood and mosquitoes was extracted using a Qiagen DNeasy Blood & Tissue Kit (Qiagen, Hilden, 24 Germany), following the manufacturer’s instructions, without the optional purification step and eluted in 20μL AE buffer to concentrate DNA. The primers C (5′-CCGCGGTAATTCCAGCTCC-3′) and J (5′-CCAACAAAAGCCGAAACGGT-3′) used for PCR were designed to amplify a 300 bp ssu rRNA fragment and specifically paired to detect the presence of trypanosomes without amplifying the dipteran ssu rRNA region, which is often co-amplified by other trypanosome primers [37, 38]. PCR protocol follows Malele et al. [38]: 95 °C for 5 min, 5 cycles of 95 °C for 1 min, 45 °C for 30 s, 65 °C for 1 min, then 35 cycles of 95 °C for 1 min, 50 °C for 30 s, 72 °C for 1 min, then finishing with a cycle of 65 °C for 30 min. PCR products were run on a 1% agarose gel using GelRed Nucleic Acid Gel Stain (Biotium, Fremont, California, USA) for 30 min at 110 V to confirm successful amplification (Biorad). PCR products were then sent to Genewiz (South Plainfield, NJ) for Sanger sequencing. Sequences were assembled and aligned using CLC Main Workbench software (Qiagen, Hilden, Germany) and analyzed using National Center for Biotechnology Information Basic Local Alignment Search Tool (NCBI BLAST). Samples with a minimum 85% identity were maintained for subsequent analyses following Altschul et al. [39]. We used *T. cruzi* DNA (source: BEI, Resources, NIAID, NIH: Genomic DNA from *Trypanosoma cruzi*, Strain G, NR-50238.) as a positive control on each gel to verify whether the conserved region of *Trypanosoma* was present and amplified.

## Results

### Field samples

We collected a total of 168 *Cx. territans* female mosquitoes during the field seasons of 2021–2023 and 2025. Trypanosomes were present in 56.5% of blood meals in these mosquitoes. By removing the head and thorax from the blood-filled abdomen in 2021 and separately testing each body section for trypanosomes, we were able to distinguish between trypanosomes obtained during feeding (presence only in the blood meal indicates ingestion) and trypanosomes that had migrated out of the gut and into the anterior tissues (head and thorax) of the mosquito from a previous blood meal (i.e., dissemination). Results yielded that 70% (*n* = 39/56) of blood meals, 4% (*n* = 2/56) of anterior tissues, and 36% (*n* = 12/33) of gravid mosquitoes in 2021 were positive for trypanosomes. It is important to note that one individual from the 2021 sample tested positive in both the blood meal and anterior tissues, and an additional sample tested positive only in the anterior tissues, but not the blood meal. To further test for dissemination in 2021, gravid females were tested to determine whether trypanosomes would be present after the blood meal was processed (i.e., not released with feces or killed during digestion) (Table 1). Mosquitoes tested during 2022, 2023, and 2025 were 24% (*n* = 6/25), 14% (*n* = 1/7), and 77% (*n* = 36/47) trypanosome-positive, respectively (Table 1).

**Table 1:** Results of trypanosome screening of *Culex territans* samples collected during this study

Year	Blood meal	Head and thorax	Gravid females	Total tested	Percentage positive
2021	39*/56 (69.6%)	2*/56 (3.6%)	12/33 (36.4%)	89	59.5%
2022	6/25 (24%)	N/A	N/A	25	24%
2023	1/7 (14%)	N/A	N/A	7	14%
2025	36/47 (76.5%)	N/A	N/A	47	77%
Total	82/135 (58.5%)	2/56 (3.6%)	12/33 (36.4%)	168	56.5%

^*^In 2021, one individual was found to be trypanosome-positive in both the anterior and posterior sections of the body and is therefore counted in both blood meal and head and thorax calculations

Blood was collected and tested for trypanosomes from a total of 199 frogs during 2022, 2023, and 2025, with an overall infection rate of 35.7% (Table 2). In 2022, 10% (*n* = 4) of *R. clamitans* and 7.7% (*n* = 2) of *R. catesbeiana* tested positive for trypanosomes. In 2023, infection rates doubled among *R. clamitans* (20%, *n* = 6) and quadrupled (30%, *n* = 6) in *R. catesbeiana*. Infection rates increased again during 2025 to 73.7% (*n* = 42) and 42.3% (*n* = 11) in *R. clamitans* and *R. catesbeiana*, respectively. Each subsequent year showed a continued rise in anuran infections, culminating in 2025, when 73.7% of *R. clamitans* (*n* = 57) and 42.3% of *R. catesbeiana* (*n* = 26) were positive for trypanosomes (Table 2).

**Table 2:** Positive anuran samples for trypanosomes

	2022	2023	2025	Total
*R. clamitans*	4/40 (10%)	6/30 (20%)	42/57 (73.7%)	52/127 (40.9%)
*R. catesbeiana*	2/26 (7.7%)	6/20 (30%)	11/26 (42.3%)	19/72 (26.3%)
Total	6/66 (9%)	12/50 (24%)	53/83 (63.8%)	71/199 (35.7%)

Two species of trypanosomes were identified from the trypanosome-positive PCR products from mosquito samples (2021–2023, *n* = 52). The most prevalent species detected was *T. ranarum* in 40 samples (76.9%), followed by *T. chattatoni* in 3 samples (0.58%); 23.1% of samples (*n* = 12) had a percentage identity below 95% and were therefore identified only to the level of genus (*Trypanosoma* spp.). Out of the blood meals that identified as *T. ranarum*, 16/40 (40%) of the mosquitoes had fed on *R. clamitans*, and 14/40 (35%) had fed on *R. catesbeiana.* The remaining 25% were from other sources or could not be identified (i.e., the blood meal had already been digested in the gravid mosquitoes). Of the three samples that contained *T. chattoni*, one of the mosquitoes had fed on *R. clamitans*, and one had fed on *R. catesbeiana*, and the third *T. chattoni* sample was from a gravid mosquito (i.e., no blood meal identification).

### Trypanosome transmission

**Figure 2:**
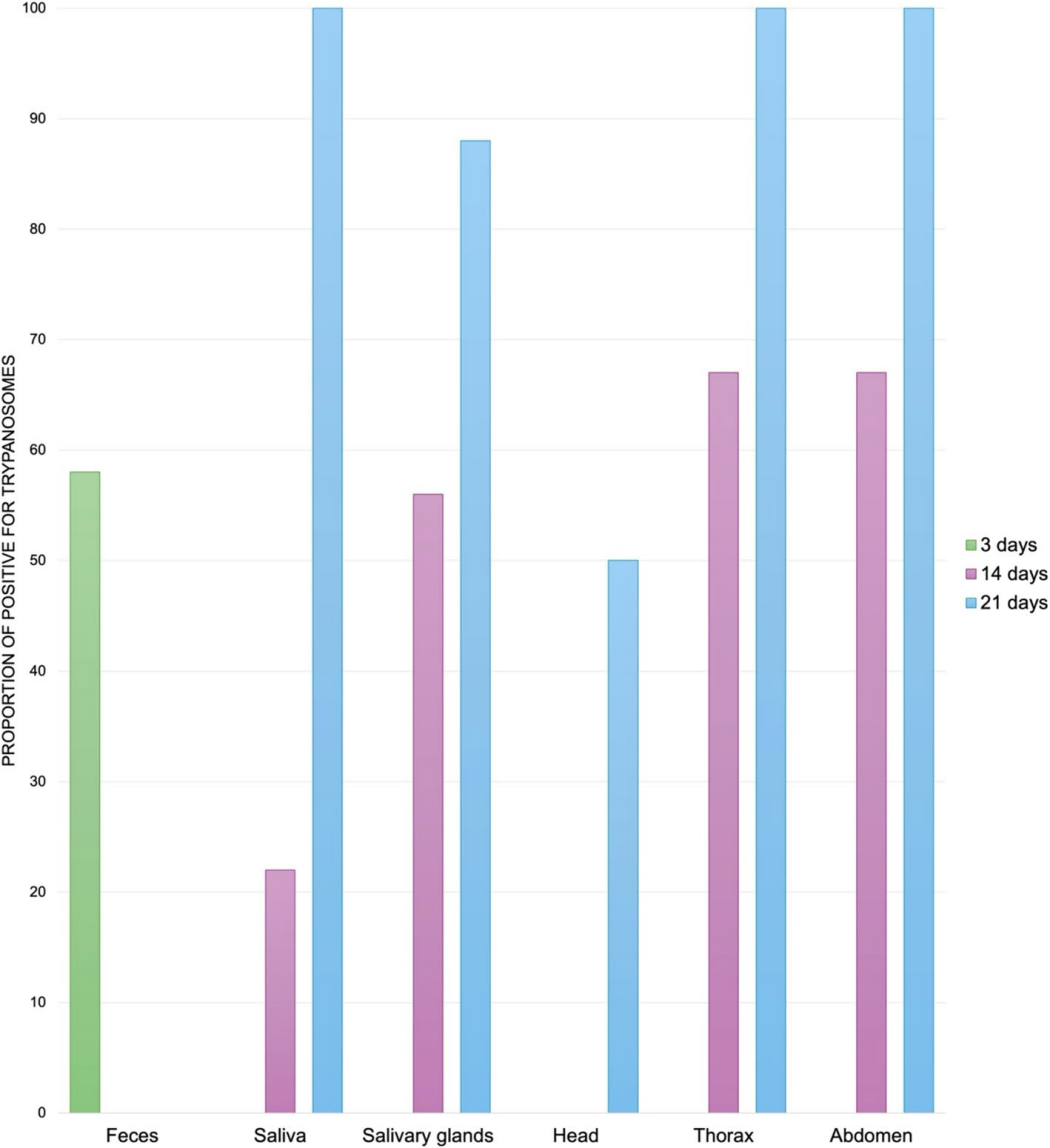
Positive detection of trypanosomes in mosquito body segments, saliva, and feces after 3 (*n* = 19), 14 (*n* = 9), and 21 (*n* = 8) days post-feeding, respectively

Results of transmission demonstrated that trypanosomes were present throughout the bodies of *Cx. territans* after 21 dpi, showing multiple potential transmission modes. Trypanosomes were found in 58% (*n* = 11) of mosquito feces 3 days after feeding (Fig. 2). At 14 days post-feeding, mosquitoes were dissected, revealing that 56% (*n* = 5) of salivary glands, 22% (*n* = 2) of saliva, and 67% (*n* = 6) of bodies (thorax and abdomen) were trypanosome-positive, but none of the heads were positive. After 21 days post-feeding, 50% (*n* = 4) of heads, 88% (*n* = 7) of salivary glands, and 100% (*n* = 8) of saliva and body samples were trypanosome positive.

## Discussion

By investigating the prevalence of trypanosomes in anurans and their potential vectors, we gain a better understanding of how trypanosomes may play a role in anuran disease ecology. This insight is crucial to elucidating the disease dynamics and pathogenic agents that threaten amphibian populations worldwide. In this study, we tested *Cx. territans* and its primary anuran hosts, *R. clamitans* and *R. catesbeiana*, for the presence of GATs and found two species circulating at MLBS in both mosquitoes and anurans. Additionally, we examined the potential for *Cx. territans* to serve as a GAT vector and furthered the knowledge of how the parasite moves through the potential vector.

Between 2021 and 2025, we confirmed the presence of two species of GATs (*T. ranarum* and *T. chattatoni*) in *Cx. territans, R. clamitans, and R. catesbeiana* populations at MLBS. This represents the first documentation of GATs in Virginia. During 2021, trypanosomes were identified in the blood meals, heads, and thoraxes of both blood-fed and gravid mosquitoes. Our results also support the parasite dissemination hypothesis within mosquitoes following an infected blood meal. We observed comparable percentages of infection among mosquito and anuran populations during 2022, 2023, and 2025, with infections increasing annually. The high prevalence of trypanosomes in both anuran (63.8%, *n* = 53/83) and mosquito (77%, *n* = 36/47) populations during 2025 may be due to the noticeably larger host and vector population sizes compared with prior years, likely caused by favorable weather conditions at MLBS (i.e., mildly warm with frequent rain; see Table S1). However, a natural cyclical disease pattern may also be a contributing factor, as many other variables can affect the epidemiology of a pathogen, including the availability of potential vectors, which we saw varied greatly from year to year with the *Cx. territans* population due to environmental fluctuations [40]. The consistently lower infection rates in *R. catesbeiana* might be due to a sampling bias or attributed to the species’ higher resistance to infection, with past studies demonstrating that subclinical infections of other anuran pathogens can, at times, be undetectable in this species [41]. Because *R. catesbeiana* is frequently used in ranaculture and the international trade of food, research, and pets [42–44], the possibility of trypanosome transmission between species and populations is of concern. Furthermore, the long time (up to 3 h) that *Cx. territans* requires to feed to repletion on *R. catesbeiana* increases the need for *Cx. territans* to feed on multiple individuals to obtain a full blood meal, further increasing the possibility of pathogen circulation [45]. If feeding is interrupted (e.g., anti-parasitic host behavior such as swatting or jumping), a single mosquito might require multiple hosts to obtain a single meal, thereby increasing the spread of pathogens within or between populations. This, combined with the hypothesis that *Cx. territans* transmits pathogens to frogs, amplifies the rate at which disease can spread within and between populations of both conspecifics and heterospecifics.

Sequence data revealed that the majority (76.9%, *n* = 40) of trypanosome samples collected during 2021–2022 were close genetic matches to previously published data for *T. ranarum*, and a smaller percentage (less than 1%, *n* = 3) with *T. chattoni*. Some samples (23.1%, *n* = 12) did not result in positive species-level identification (i.e., above 95%). The taxonomic uncertainty of these results may reflect the short (300 bp) sequence fragment used for identification and could be overcome via whole genome sequencing. Alternatively, it is possible that these samples represent co-infections or previously undescribed species, as discussed by Spordareva et al. [11]. However, even without the exact species identification on all samples, it is clear that GATs are present at MLBS. Trypanosomes can vary greatly in prevalence, due to location, season, host, and species [8, 46]. Bartlett-Healy et al. [8] collected blood-fed *Cx. territans* in New Jersey over the course of 3 years and found GATs in the blood meals April–August, with a peak in June, temporally corresponding with the active season of *Cx. territans* and its hosts [47]. They also found variations of prevalence based on location, ranging 6–100% of infected *Cx. territans* blood meals. Our study showed a similarly broad range (14–76.5%). The GAT species can change on the basis of the host species as well. Barta and Desser [46] found *T. ranarum* prevalence in *R. catesbeiana* and *R. clamitans* to be fairly low (4% and 10%, respectively), while *T. rotatorium* had higher prevalence (52% and 43%). This differs greatly from our findings, as we found a higher prevalence of *T. ranarum* in blood meals derived from both *R. clamitans* and *R. catesbeiana*. The predominant species varies with location as well, as Bartlett-Healy et al. [8] saw a low prevalence of *T. rotatorium* in New Jersey, USA, compared with Barta and Desser in Ontario, Canada [46]. Woo and Bogart [48] found variations in species in locations along the eastern US states, focusing on GATs found in tree frogs, and even the location that Woo and Bogart tested that is closest to our study site (West Virginia) differs greatly in species compared with our site.

To further understand the potential of mosquitoes as vectors of anuran trypanosomes, we investigated the potential modes of transmission of GATs by *Cx. territans*. Whereas *Cx. territans* has been suspected of being a GAT vector, their vector status has not yet been confirmed [8, 49]. Desser et al. [19] observed development of *T. rotatorium* in the midgut of *Cx. territans*, suggesting that this mosquito may be a competent vector; however, development does not guarantee transmission capabilities. This is supported by the observation that, although *T. rotatorium* develops in *Cx. territans*, the stage found by Desser et al. [49] in the hindgut of the mosquito 100 h post-infection is not infectious to its anuran hosts. Furthermore, Desser et al. [49] only identified trypanosomes in the hindgut, which is how *T. cruzi* is transmitted by their kissing bug vectors (i.e., via the insects’ feces) [27]. Our results demonstrate that parasite dissemination through the body of the mosquito can take longer than 100 h, suggesting other possibilities for transmission. There are more than 27 species of GATs [11], and developmental patterns may differ within the mosquito, but how these patterns vary requires further investigation.

Multiple modes of transmission exist that would allow *Cx. territans* to transmit trypanosomes. We tested: (1) the saliva and the salivary glands to determine whether they can transmit parasites such as tsetse flies [27]; (2) the head to test whether the trypanosomes cling to the esophagus as in *Cx. quinquefasciatus* [28, 29]; (3) the body to determine whether they could transmit the trypanosomes in the same way *Hepatozoon* parasites are spread [25, 26]; and (4) the feces to determine whether they could transmit the parasites in the same way kissing bugs do [27]. We identified trypanosomes in 58% of the mosquito feces samples, indicating presence in the hindgut. Interestingly, the presence of trypanosomes in gravid mosquitoes, as well as presence in the head and thorax (and not in the blood meal) in the 2021 field-caught blood-fed mosquitoes, indicates that the trypanosomes may not be released only through defecation, but instead may migrate to the anterior portion of the body. However, they do not appear to migrate into all anterior tissues within 14 dpi, since the head tissues were not infected at that point. Yet, salivary glands and saliva of some of the mosquitoes were infected at 14 dpi, which is likely due to the location of the salivary glands in the thorax, not the head. The slow migration through the tissues of the mosquito seen in the results implies that the parasite development and dissemination may take up to 21 days. Because of the migration of trypanosomes to the saliva, we suggest that, if *Cx. territans* is a vector of trypanosomes, the most likely mode of transmission is through saliva while feeding on an anuran host. It seems unlikely that direct transmission of trypanosomes via the feces is significant in this system because mosquitoes usually take 2–3 days to digest and defecate after a blood meal, at which point they would no longer be in contact with the host, and the parasite would likely not develop to the infectious stage at that time [49]. However, it is worth noting that, while uncommon, especially at the lower temperatures of *Cx. territans*’ hosts, mosquitoes usually produce fluid during feeding to concentrate the blood meal [50, 51] (personal observation). It is thus possible that trypanosomes may be transmitted in this way if they migrated to the Malpighian tubules or if gut content is released at the same time as observed in other mosquito species [52]. Further studies are needed to determine whether trypanosomes are present in the urine during the secondary feeding following initial infection. Ingestion by the host is another potential mode of transfer of trypanosomes, since some parasites remain in the mosquito body and continue to develop, similar to the transmission of *Hepatozoon* parasites [25]. With consideration given to these potential routes, however, we acknowledge the limitations of relying solely on molecular methods to determine the progression of the parasite through the mosquito body. The developmental stage of the parasite is an important factor in transmission, and future studies will include histological studies to determine whether the infectious stage is found in the saliva or throughout the body of the mosquito.

## Conclusions

This study confirmed the presence of two species of GATs (*T. ranarum* and *T. chattatoni*) in both the *Cx. territans* and the anuran populations in SW Virginia and adds to the growing knowledge regarding GATs and the transmission of these parasites. Results suggest that trypanosomes are most likely transmitted via saliva during biting in *Cx. territans*. A second route of transmission, ingestion, is supported by our observations of parasite migration through the mosquito occurring over a 21-dpi period. Future research is required to determine whether the trypanosomes present in the saliva are in the infectious stage. Moreover, determining the length of time the trypanosomes remain in the mosquitoes is an important question to answer, considering a long incubation period will affect the transmission window of GATs to their amphibian hosts.

## Supplementary Information

Supplementary Material 1.

## Data Availability

The data supporting the findings of this study are available within the article.

## Abbreviations

*Cx*.: *Culex*
*R*.: *Rana*
*T*.: *Trypanosoma*
GATs: Giant Anuran Trypanosomes
MLBS: Mountain Lake Biological Station
dpi: Days post-infection
PCR: Polymerase chain reaction
PBS: Phosphate buffered saline

## References

[1] Brunner JL, Storfer A, Gray MJ, Hoverman JT. Ranavirus ecology and evolution: from epidemiology to extinction. Cham: Springer; 2015.

[2] Gray MJ, Miller DL, Hoverman JT. Ecology and pathology of amphibian ranaviruses. Dis Aquat Organ. 2009;3:243–66. 10.3354/dao02138 20099417

[3] Re:wild, Synchronicity Earth, IUCN SSC Amphibian Specialist Group. State of the World’s Amphibians: The Second Global Amphibian Assessment. Texas, USA: Re:wild; 2023.

[4] Chen Z, Cao YS, Dong MS, Li WB. Human activities and climate change are the main factors of amphibian extinction. Global Ecology and Conservation. 2025 Jul 16:e03747.

[5] Collins JP. Amphibian decline and extinction: what we know and what we need to learn. Dis Aquat Organ. 2010;92:93–9. 21268970 10.3354/dao02307

[6] Robert J. Emerging ranaviral infectious diseases and amphibian decline. Diversity. 2010;2:314–30.

[7] Stuart SN, Chanson JS, Cox NA, Young BE, Rodrigues AS, Fischman DL, et al. Status and trends of amphibian declines and extinctions worldwide. Science. 2004;306:1783–6. 15486254 10.1126/science.1103538

[8] Bartlett-Healy K, Crans W, Gaugler R. Vertebrate hosts and phylogenetic relationships of amphibian trypanosomes from a potential invertebrate vector, *Culex territans* Walker (Diptera: Culicidae). J Parasitol. 2009;95:381–7. 18850768 10.1645/GE-1793.1

[9] Chimelli L, Scaravilli F. Trypanosomiasis. Brain Pathol. 1997;7:599–611. 9034568 10.1111/j.1750-3639.1997.tb01077.x PMC8098396

[10] Martin DS, Wright AD, Barta JR, Desser SS. Phylogenetic position of the giant anuran trypanosomes *Trypanosoma chattoni, Trypanosoma fallisi, Trypanosoma mega, Trypanosoma neveulemairei,* and *Trypanosoma ranarum* inferred from 18s rRNA gene sequences. J Parasitol. 2002;88:566–71. 12099428 10.1645/0022-3395(2002)088[0566:PPOTGA]2.0.CO;2

[11] Spodareva VV, Grybchuk-Ieremenko A, Losev A, Votýpka J, Lukeš J, Yurchenko V, et al. Diversity and evolution of anuran trypanosomes: insights from the study of European species. Parasit Vectors. 2018;11:447. 30071897 10.1186/s13071-018-3023-1 PMC6090815

[12] da.Ferreira S, daCosta JIAP, Ramirez D, Roldan JA, Saraiva D, da.Founier SGF, et al. Anuran trypanosomes: phylogenetic evidence for new clades in Brazil. Syst Parasitol. 2015;91:63–70. 25862033 10.1007/s11230-015-9558-z

[13] Leal DD, O’dwyer LH, Ribeiro VC, Silva RJ, Ferreira VL, Rodrigues RB. Hemoparasites of the genus *Trypanosoma* (Kinetoplastida: Trypanosomatidae) and hemogregarines in anurans of the São Paulo and Mato Grosso do Sul States-Brazil. An Acad Bras Ciênc. 2009;81:199–206. 19488624 10.1590/s0001-37652009000200006

[14] Sailasuta A, Satetasit J, Chutmongkonkul M. Pathological study of blood parasites in rice field frogs, *Hoplobatrachus rugulosus* (Wiegmann, 1834). Vet Med Int. 2011;2011:850568. 21918731 10.4061/2011/850568 PMC3171767

[15] Bardsley JE, Harmsen R. The trypanosomes of anura. Adv Parasitol. 1973;1:1–73. 10.1016/s0065-308x(08)60184-0 4210097

[16] Martin DS, Desser SS. Development of *Trypanosoma fallisi* in the leech, *Desserobdella picta*, in toads (*Bufo americanus*), and in vitro: a light and electron microscopic study. Parasitol Res. 1991;77:18–26. 1994366 10.1007/BF00934379

[17] Siddall ME, Desser SS. Alternative leech vectors for frog and turtle trypanosomes. J Parasitol. 1992;78:562–3. 1597811

[18] Johnson RN, Young DG, Butler JF. Trypanosome transmission by *Corethrella wirthi* (Diptera: Chaoboridae) to the green treefrog, *Hyla cinerea* (Anura: Hylidae). J Med Entomol. 1993;30:918–21. 8254641 10.1093/jmedent/30.5.918

[19] Desser SS, McIver SB Ryckman A. *Culex territans* as a potential vector of *Trypanosoma rotatorium*. I.Development of the flagellate in the mosquito. J Parasitol. 1973. 353-358. 10.2307/3278833. 4696582

[20] Hamer GL, Kitron UD, Brawn JD, Loss SR, Ruiz MO, Goldberg TL et al. *Culex pipiens* (Diptera: Culicidae): abridge vector of West Nile virus to humans. J Med Entomol. 2008. 45:125–8. 18283952 10.1603/0022-2585(2008)45[125:cpdcab]2.0.co;2

[21] Molaei G, Andreadis TG, Armstrong PM, Anderson JF, Vossbrinck CR. Host feeding patterns of Culex mosquitoesand West Nile virus transmission, northeastern United States. Emerg Infect Dis. 2006. 12(3):468-474. 16704786 10.3201/eid1203.051004 PMC3291451

[22] Molaei G, Andreadis TG, Armstrong PM, Bueno R, Dennett JA, Real SV et al. Host feeding pattern of *Culex quinquefasciatus* (Diptera: Culicidae) and its role in transmission of West Nile virus in Harris County. Texas Am J TropMed Hyg. 2007. 77:73–81. 17620633

[23] Rajagopalan PK, Kazmi SJ, Mani TR. Some aspects of transmission of *Wuchereria bancrofti* and ecology of the vector *Culex pipiens fatigans* in Pondicherry. Indian J Med Res 66:200–215 (1977) 336534

[24] Benach JL. Observations of a colony of *Culex territans* Walker. Proc. N J Mosq. Exterm. Assoc 57:1970. 70–76.

[25] Ferguson LV, Smith TG. Reciprocal trophic interactions and transmission of blood parasites between mosquitoes and frogs. Insects. 2012;3:410–23. 26466534 10.3390/insects3020410 PMC4553601

[26] Smith TG. The genus *Hepatozoon* (apicomplexa: adeleina). J Parasitol. 1996;1:565–85. 8691364

[27] Vickerman K. Developmental cycles and biology of pathogenic trypanosomes. Br Med Bull. 1985;41:105–14. 3928017 10.1093/oxfordjournals.bmb.a072036

[28] Volf P, Hajmova M, Sadlova J, Votypka J. Blocked stomodeal valve of the insect vector: similar mechanism of transmission in two trypanosomatid models. Int J Parasitol. 2004;34:1221–7. 15491584 10.1016/j.ijpara.2004.07.010

[29] Votýpka J, Szabova J, Radrova J, Zídková L, Svobodova M. *Trypanosoma culicavium* sp. nov., an avian trypanosome transmitted by *Culex *mosquitoes. Int J Syst Evol Microbiol. 2012;62:745–54. 21515704 10.1099/ijs.0.032110-0

[30] Fermin G. Host range, host–virus interactions, and virus transmission. Viruses. 2018;30:101.

[31] Reinhold JM, Halbert E, Roark M, Smith SN, Stroh KM, Siler CD et al. The role of *Culex territans* mosquitoes inthe transmission of *Batrachochytrium dendrobatidis* to amphibian hosts. Parasit Vectors. 2023. 16:424. 37974288 10.1186/s13071-023-05992-x PMC10655354

[32] Darsie Jr RF, Ward RA. Identification and geographical distribution of the mosquitoes of North America, north of Mexico. Walter Reed Army Inst of Research Washington DC. 1981.

[33] Yuan ZY, Zhou WW, Chen X, Poyarkov Jr NA, Chen HM et al. Spatiotemporal diversification of the true frogs (genus *Rana*): a historical framework for a widely studied group of model organisms. Syst Biol. 2016. 65:824–42. 27288482 10.1093/sysbio/syw055

[34] AmphibiaWeb. University of California, Berkeley, CA, USA. 2005. https://amphibiaweb.org. Accessed 15 Oct 2025.

[35] Martoff BS. Territoriality in the green frog, *Rana clamitans*. Ecology. 1953;34:165–74.

[36] Forzán MJ, Heatley J, Russell KE, Horney B. Clinical pathology of amphibians: a review. Vet Clin Pathol. 2017;46:11–33. 28195641 10.1111/vcp.12452

[37] Maslov DA, Lukeš J, Jirku M, Simpson L. Phylogeny of trypanosomes as inferred from the small and large subunit rRNAs: implications for the evolution of parasitism in the trypanosomatid protozoa. Mol Biochem Parasitol. 1996;75:197–205. 8992318 10.1016/0166-6851(95)02526-x

[38] Malele I, Craske L, Knight C, Ferris V, Njiru Z, Hamilton P et al. The use of specific and generic primers to identify trypanosome infections of wild tsetse flies in Tanzania by PCR. Infect Genet Evol. 2003. 3:271–9. 14636688 10.1016/s1567-1348(03)00090-x

[39] Altschul SF, Gish W, Miller W, Myers EW, Lipman DJ. Basic local alignment search tool. J Molec Biol. 1990;215:403–10. 2231712 10.1016/S0022-2836(05)80360-2

[40] Rogers DJ, Williams BG. Monitoring trypanosomiasis in space and time. Parasitology. 1993;106:S77-92. 8488074 10.1017/s0031182000086133

[41] Brunner JL, Olson AD, Rice JG, Meiners SE, Le Sage MJ, Cundiff JA et al. Ranavirus infection dynamics andshedding in American bullfrogs: consequences for spread and detection in trade. Dis Aquat Organ. 2019. 135:135–50. 31392966 10.3354/dao03387

[42] Altmann MC, Kolby JE. Trends in US imports of amphibians in light of the potential spread of chytrid fungus, *Batrachochytrium dendrobatidis* (*Bd*), and implications for conservation. J Int Wildl Law Policy. 2017;20:226–52.

[43] Schlaepfer MA, Hoover C, Dodd CK. Challenges in evaluating the impact of the trade in amphibians and reptiles on wild populations. Bioscience. 2005;55:256–64.

[44] Schloegel LM, Picco AM, Kilpatrick AM, Davies AJ, Hyatt AD, Daszak P. Magnitude of the US trade in amphibians and presence of *Batrachochytrium dendrobatidis* and ranavirus infection in imported North American bullfrogs (Rana catesbeiana). Biol Conserv. 2009;142:1420–6.

[45] Reinhold J. Some like it cold: interactions between the northern frog biting mosquito, *Culex territans* (Walker 1856), and its amphibian hosts. Blacksburg: Virginia Tech; 2023.

[46] Barta JR, Desser SS. Blood parasites of amphibians from Algonquin Park. Ontario J Wildl Dis. 1984;20:180–9. 6492319 10.7589/0090-3558-20.3.180

[47] Bartlett-Healy K, Crans W, Gaugler R. Temporal and spatial synchrony of *Culex territans* (Diptera: Culicidae) with their amphibian hosts. J Med Entomol. 2008;45:1031–8. 19058626 10.1603/0022-2585(2008)45[1031:tassoc]2.0.co;2

[48] Woo PT, Bogart JP. *Trypanosoma* spp (Protozoa: Kinetoplastida) in Hylidae (Anura) from eastern North America,with notes on their distributions and prevalences. Can J Zool. 1984. 62(5): 820-824. 10.1139/z84-119.

[49] Desser SS, McIver SB, Jez D. Observations on the role of simuliids and culicids in the transmission of avian and anuran trypanosomes. Int J Parasitol. 1975;5:507–9. 1080482 10.1016/0020-7519(75)90041-7

[50] Beyenbach KW, Petzel DH. Diuresis in mosquitoes: role of a natriuretic factor. Physiology. 1987;2:171–5.

[51] Reinhold JM, Shaw R, Lahondère C. Beat the heat: *Culex quinquefasciatus* regulates its body temperature during blood feeding. J Therm Biol. 2021;96:102826. 33627266 10.1016/j.jtherbio.2020.102826

[52] Lahondère C, Lazzari CR. Mosquitoes cool down during blood feeding to avoid overheating. Curr Biol. 2012;22:40–5. 22177900 10.1016/j.cub.2011.11.029

