# Supplementary material for: Culex territans mosquitoes as a vector of Giant Anuran Trypanosomes
Source: Parasit Vectors. 2026 Apr 21;19:240. doi: 10.1186/s13071-026-07399-w (PMC13231525; doi:10.1186/s13071-026-07399-w)
Supplement: Supplementary file 1 — Supplementary Material 1. [file 13071_2026_7399_MOESM1_ESM.docx]

**Table S1**: Monthly maximum, minimum, and mean temperatures (°F) (top) and total precipitation (inches) (bottom) at Mountain Lake Biological Station, May–August 2021–2023 and 2025 (Source: Mountain Lake Biological Station weather station: KVANEWPO68).

| **Month**  **Year** | **MAY** | **JUNE** | **JULY** | **AUGUST** |
| --- | --- | --- | --- | --- |
| **2021** | 77 / 32.9 (56.3)  1.59 | 78.3 / 41.8 (62.9)  4.21 | 81.8 / 49.6 (66)  2.49 | 83.8 / 52.5 (66.6)  5.79 |
| **2022** | 81.4 / 34.4 (57.6)  8.24 | 85.1 / 39.8 (63.5)  2.75 | 83.3 / 49.2 (67)  0.58 | 80.8 / 51.2 (64.7)  0.06 |
| **2023** | 72.4 / 30.3 (52.9)  2.61 in | 77.4 / 40.1 (58.6)  1.35 | 82.6 / 50 (66.2)  0.06 | 81.8 / 47.5 (64.4)  0.45 |
| **2025** | 75.3 / 37.8 (54.4)  10.3 | 85.6 / 37.9 (66)  3.35 | 83.4 / 57. 2 (69)  7.29 | 80 43 (62)  4.02 |
